# A subcellular cookie cutter for spatial genomics in human tissue

**DOI:** 10.1007/s00216-022-03944-5

**Published:** 2022-03-02

**Authors:** Alexander G. Bury, Angela Pyle, Fabio Marcuccio, Doug M. Turnbull, Amy E. Vincent, Gavin Hudson, Paolo Actis

**Affiliations:** 1 Wellcome Centre for Mitochondrial Research, Medical School, Newcastle University, Newcastle-upon-Tyne, NE2 4HH UK; 2 Biosciences Institute, Medical School, Newcastle University, Newcastle-upon-Tyne, NE2 4HH UK; 3 Bragg Centre for Materials Research, University of Leeds, Leeds, LS2 9JT UK; 4 School of Electronic and Electrical Engineering, University of Leeds, Leeds, LS2 9JT UK; 5 Translational and Clinical Research Institute, Medical School, Newcastle University, Newcastle-upon-Tyne, NE2 4HH UK

**Keywords:** Subcellular, Genomics, SICM, Isolation, Mitochondria, Organelle

## Abstract

Intracellular heterogeneity contributes significantly to cellular physiology and, in a number of debilitating diseases, cellular pathophysiology. This is greatly influenced by distinct organelle populations and to understand the aetiology of disease, it is important to have tools able to isolate and differentially analyse organelles from precise location within tissues. Here, we report the development of a subcellular biopsy technology that facilitates the isolation of organelles, such as mitochondria, from human tissue. We compared the subcellular biopsy technology to laser capture microdissection (LCM) that is the state-of-the-art technique for the isolation of cells from their surrounding tissues. We demonstrate an operational limit of  >20 µm for LCM and then, for the first time in human tissue, show that subcellular biopsy can be used to isolate mitochondria beyond this limit.

**Graphical abstract:**

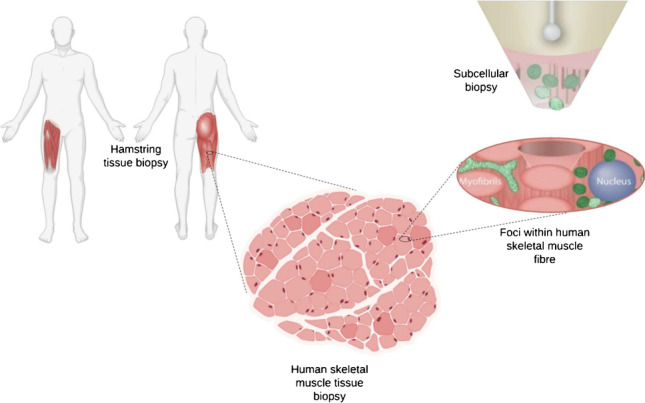

## Introduction

Inter-tissue and inter-cellular heterogeneity is a known contributor to a number of human diseases including cancer [1–3]; cardiovascular disease [4, 5]; metabolic disease [6–9]; and neurodegeneration, neurodevelopmental disorders, and pathological ageing [10–13]. Yet, evaluating heterogeneity at the tissue and cellular level can often mask subtle subcellular and organelle heterogeneity [14]. In addition to morphological and functional heterogeneity, exhibited by other organelles, mitochondria additionally show genetic heterogeneity, owing to their own multi-copy genome [9, 15]. The mitochondrial genome (mtDNA) exists as uniform wild-type molecules at birth, in healthy individuals—termed homoplasmy, but de novo mutations give rise to a mixture of wild-type and mutant mtDNA molecules—termed heteroplasmy [16]. Whilst low level heteroplasmy is well tolerated, the accumulation and spread of mutant mtDNA molecules in excess of a threshold level can lead to impaired oxidative phosphorylation that often culminates in mitochondrial disease [16, 17]. The mechanism behind this process, termed clonal expansion, is not fully understood. Investigating clonal expansion at the subcellular level may advance our understanding of the mechanisms behind it and help improve characterisation of mitochondrial disease [18, 19]. More generally, better understanding the physiological (and pathophysiological) relevance of intracellular organelle heterogeneity with subcellular precision would likely aid effective diagnosis and treatment of disease [9, 20–22]. Here, we present the subcellular “cookie cutter”—capable of cutting and isolating microscale cylinders of tissue from distinct foci, with nanoscale precision.

To take full advantage of “single-cell multiomics” [23, 24], nanoprobe-based technologies can circumvent common challenges associated with investigating subcellular molecules, including the requirement to permeabilise cells and implementation of complex biochemical reactions which do not allow, for example, the analysis of intracellular organelles [25–29]. Nanoprobe technologies are typically integrated with scanning probe microscopy [30–32] or atomic force microscopy [33–36] to enable their automated positioning in and around cells with nanometer precision. Increasingly, pipette-based nanoprobes are being used to investigate the electrochemistry, intracellular signalling, as well as the detection and delivery of single molecules with subcellular precision [37–41]. The comparatively small probe size also enables sampling from live cells with minimal impact on cell viability or the cellular environment [42].

In 2014, Actis and colleagues developed the nanobiopsy technology which utilises a nanopipette containing an organic solvent to aspirate mRNA and mitochondria within the cytoplasm of cultured fibroblasts [27]. This methodology relies on electrowetting, a process where a liquid–liquid interface is manipulated by the application of a voltage to aspirate a target from the cytoplasm of a living cell [30, 31, 42]. More recently, fluid force microscopy (FFM), dielectrophoretic nanotweezers, and nanopipettes have successfully been used to sample cytoplasmic proteins and nucleic acids [27, 33–36] from cells in culture. However, none of these technologies has been applied to the study of tissue samples that are routinely used for clinical and molecular pathology [8, 37, 38]. Here, we aimed to determine if nanobiopsy could be adapted for sampling from human tissue samples.

**Fig. 1:**
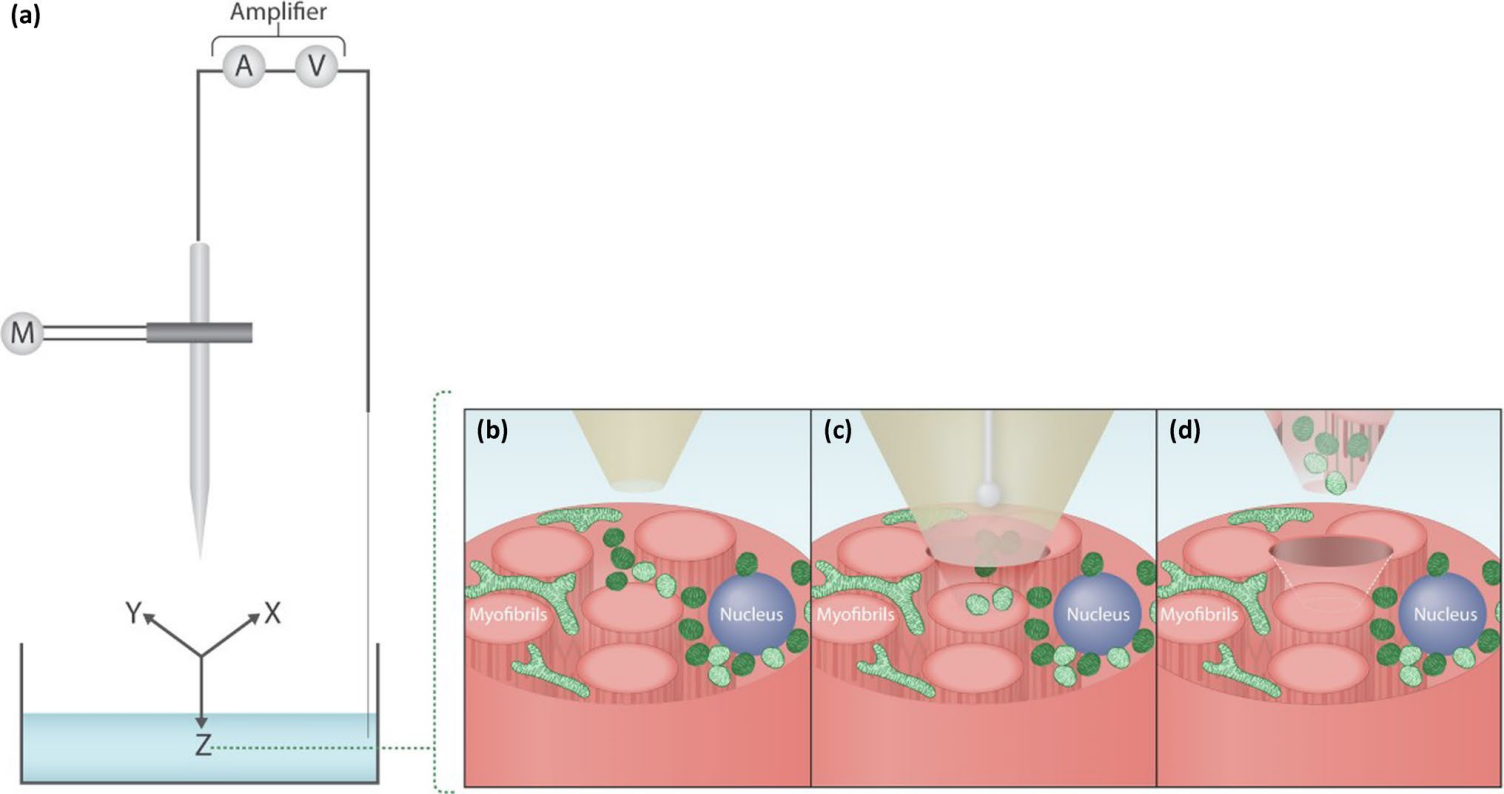
(**a**) Subcellular biopsy in skeletal muscle. A micropipette is incorporated within a scanning ion conductance microscope to enable its automated positioning. (**b**) A micropipette is immersed within an aqueous bath in which the sample of interest is placed, such as skeletal muscle tissue. The micropipette can be positioned within 1 µm of the skeletal muscle fibre of interest. (**c**) The micropipette then penetrates the tissue by a predefined distance to perform the subcellular biopsy. (**d**) The micropipette is then retracted and the biopsied contents are deposited into a collection vessel

Electrowetting is necessary for the sampling from cultured cells because manipulating the liquid–liquid interface creates a force that draws the cytoplasm into the pipette tip [42]. Our data shows that the nanopipette effectively acts as a subcellular “cookie cutter” removing the need for electrowetting (Fig. 1). This is important because electrowetting relies on the use of a toxic organic solvent, 1,2-dichloroethane, that can potentially affect the quality of downstream molecular analyses [43]. Using laser capture microdissection (LCM), the most commonly utilised approach for studying single cells from tissue samples [8, 10, 44–46, 65], as a comparator, we show that an adaptation of nanobiopsy, subcellular biopsy, has the potential to go beyond conventional methodological limits.

## Materials and methods

### Skeletal muscle tissue biopsies

Skeletal muscle tissue biopsies (*n* = 14) were obtained from volunteers undergoing anterior cruciate ligament (ACL) surgery at the Freeman General Hospital (Newcastle, UK). Consent and ethical approval were granted by The Newcastle and North Tyneside Local Research Ethics Committee (LREC: 12/NE/0395). All research was carried out in correspondence with the Human Tissue Act (HTA) (2004) with HTA licensing and material transfer agreements between Newcastle University and the University of Leeds. Biopsied tissue was snap frozen in liquid nitrogen cooled isopentane and mounted transversely in OCT mounting medium. Cryosections were collected onto slides (Agar Scientific Ltd, Essex, UK), air-dried for 1 h, and stored at − 80 °C prior to staining.

### Immunofluorescence staining

Briefly, tissue sections were fixed with 4% PFA (Sigma-Aldrich, MO, USA) and permeabilised using a methanol gradient (Thermo Fisher, Paisley, UK). A protein block (10% normal goat serum, Vector, Peterborough, UK) was carried out at room temperature for 1 h and endogenous biotin blocking (Avidin D/Biotin, Vector) was carried out applying each solution for 15 min at room temperature, to minimise non-specific staining. Primary and secondary antibodies were applied overnight and for 2 h, respectively, at 4 °C in a humidified chamber. Primary antibodies: anti-mitochondrial cytochrome c oxidase I (MTCOI; ab14705, Abcam, Cambridge, UK), anti- NADH dehydrogenase subunit B8 (NDUFB8; ab110242, Abcam); anti-Voltage Dependent Anion Channel 1 (VDAC1; ab14734, Abcam); 4,6‐diamidino‐2‐phenylindole nuclear stain (DAPI; Sigma, St. Louis, MO). Secondary antibodies (all Life Technologies): anti-mouse IgG2a- 488 (S21131), biotinylated anti‐mouse IgG1 (S32357), streptavidin‐647 (S21374) and anti-mouse IgG2b-546 (A21143).

### Laser capture microdissection (LCM) and tissue lysis

Isolation of single- and subcellular muscle fibre dissections was performed using a PALM LCM system (Zeiss, Oberkochen, DE). Microdissected samples were collected in inverted 0.2-ml microfuge tubes containing 15 µl of lysis buffer (0.5 M Tris–HCl, 0.5% Tween 20, 1% proteinase K at pH 8.5), as previously described [47]. Samples were centrifuged and then incubated at 55 °C for 3 h then at 95 °C for 10 min, using a thermal cycler (Applied Biosystems), to ensure efficient lysis of single cells and mitochondria to yield mtDNA.

### Fabrication and analysis of micropipettes

Micropipettes were fabricated from quartz glass capillaries (QF100-50–7.5, Sutter Instrument, Novato, CA) using a P-2000 micropipette laser puller (World Precision Instruments, Sarasota, FL). An Ag/AgCl wire was inserted in the micropipette to act as the working electrode and another Ag/AgCl wire was immersed in a 1 × PBS bath (Oxoid Ltd, Thermo Fisher, UK), acting as the reference electrode. Current–voltage measurements are performed using a MultiClamp 700B patch-clamp amplifier (Molecular Devices, Sunnyvale, CA). The signal was filtered using a Digidata 1550B digitizer, with a low-pass filter at 10 kHz, and signal recording was performed using the pClamp 10 software (Molecular Devices), at a rate of 100 kHz. For electrochemical analysis experiments, the micropipettes were filled with 1 × PBS. For electrowetting experiments, the micropipettes were filled with a solution of 10 mM tetrahexylammonium tetrakis(4-chlorophenyl)borate (THATPBCl) salt in 1,2-dichloroethane (1,2-DCE).

### Scanning electron microscopy (SEM)

A Vega 3 scanning electron microscope (Tescan, Brno, CZ) was used to image and determine the micropipette geometry and aperture size. Micropipettes were sputter coated with a 5-nm layer of gold before being mounted onto a sample holder. Imaging parameters were as follows: acceleration voltage: 8 kV; beam intensity: 6–8; working distance: 15–30 mm; magnification: × 10 k–60 k.

### Scanning ion conductance microscopy (SICM)

The SICM setup was comprised of an Axon MultiClamp 700B amplifier, MM-6 micropositioner (Sutter Instrument, Novato, CA) and a P-753 Linear actuator (Physik Instrumente, Irvine, CA) attached to the pipette holder to allow precise, three-dimensional movement of the micropipette (Fig. 1a). SICM software was used to control the positioning and topographical scanning capabilities of the SICM system (ICAPPIC, London, UK). An Eclipse Ti2 confocal microscope (Nikon Instruments Inc., Melville, NY) and broad-spectrum LED illumination system (pE-300 CoolLED, Andover, USA) were used for bright-field (BF) and immunofluorescence (IF) visualisation of mitochondria and myonuclei, in skeletal muscle fibres, to ensure efficient biopsy. The SICM system is used to automatically approach the skeletal muscle tissue, moving the micropipette just above the region of interest (Fig. 1b), which is then inserted into the skeletal muscle fibre through manual control (Fig. 1c). Following successful biopsy of mitochondria, the micropipette was retracted and the tip snapped into a 0.2-ml microfuge tubes containing lysis buffer (Fig. 1d) as described in the “Laser capture microdissection (LCM) and tissue lysis” section.

### Triplex quantitative PCR (qPCR)

To quantify total mtDNA CN, the abundance of the *MTND1* probe (mtDNA minor arc) was measured, indicative of total mtDNA [48]. The *D Loop* probe, corresponding to another highly conserved region of the mitochondrial genome, was used as a comparator in case of rare instances of minor arc deletions that could affect accurate CN assessment [49]. Samples were plated in triplicate and each qPCR run was repeated, to control for inter-run variation.

### Statistical analysis

The variance in the median between electrowetting and non-electrowetting groups, and each of electrowetting and non-electrowetting groups against non-biopsy controls (NBC), was compared using Mann–Whitney *U* tests. The correlation between mtDNA CN and LCM dissection or subcellular biopsy size was tested using linear regression. Statistical significance was set as *p* < 0.05. Statistical analyses were performed and graphs produced using GraphPad Prism version 5.00 for Windows (GraphPad Software, San Diego, CA).

## Results

### Working range of laser capture microdissection (LCM)

**Fig. 2:**
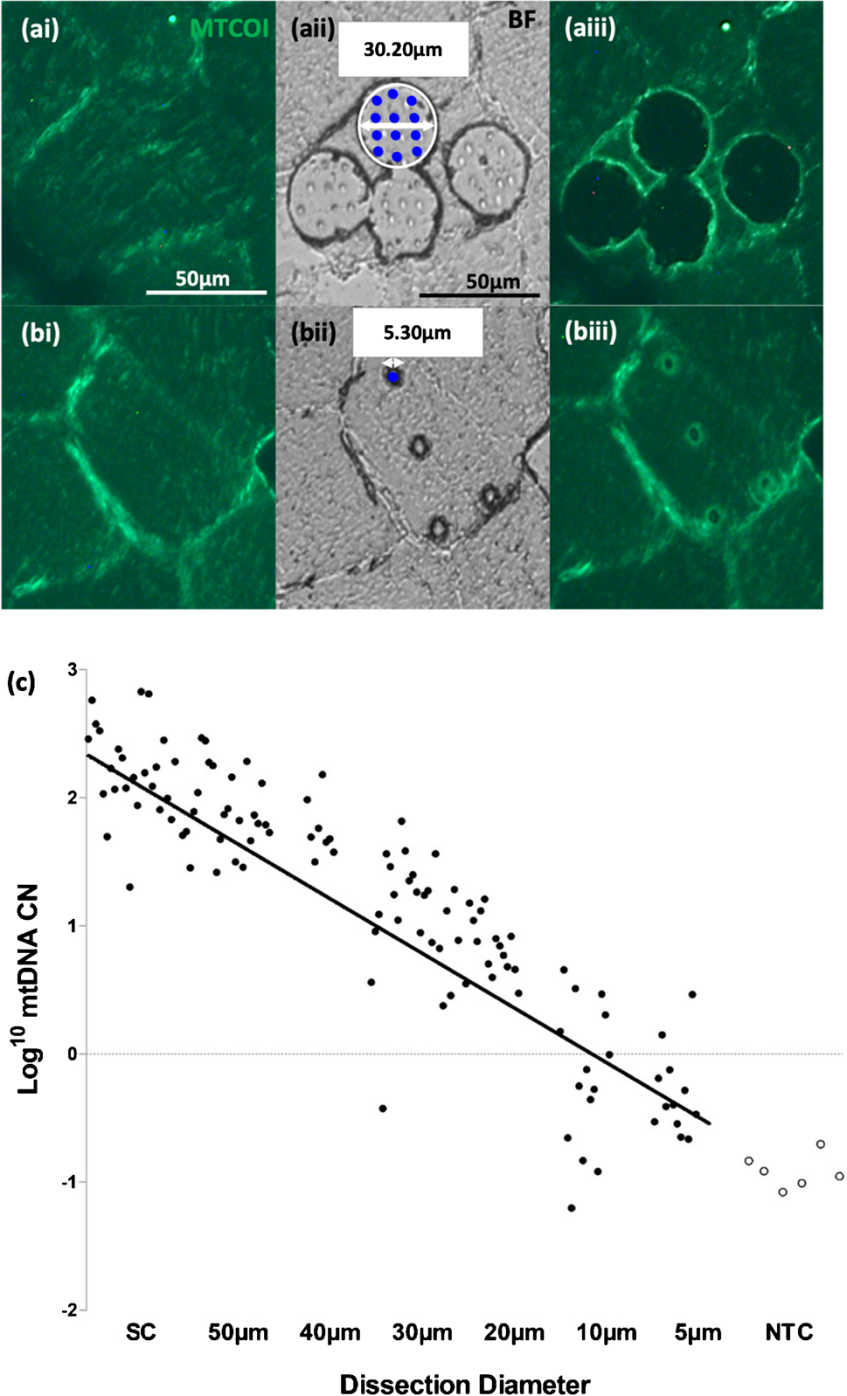
Working limit of LCM. Panels (**a**) and (**b**) correspond to biopsies taken using LCM with dissection diameters of 30 µm (**a**) and 5 µm (**b**), respectively. The first two panels (left to right) correspond to pre-biopsy immunofluorescent (IF) images used to identify the area of interest (ai, bi). The middle panels show the BF post-cut images with the delineated cutting area outlined in white (aii) and laser pulse markers, shown as blue dots (aii, bii). The right panels correspond to post-dissection IF images showing the indentations made by the cutting laser and laser pulses and the relative change in fluorescence indicating successful isolation of tissue (aiii, biii). CN/µl values (log transformed, *y*-axis) were plotted against LCM dissection size (µm). Regression analysis demonstrated a significant relationship between LCM dissection size and CN/µl (*n* = 14; Spearman correlation = 0.91, *p* < 0.05). A greater proportion of dissections under 20 µm in diameter corresponded to less than 1 mtDNA copies (< 0 log transformed CN/µl), compared with dissections larger than 20 µm (**c**). All dissections taken corresponded to their labelled diameter ± 10%, except 5 µm (± 20%). All data points in black correspond to technical triplicates of lysate samples taken using LCM. All data points in white correspond to technical triplicates of non-template controls.

LCM is the most established technique for isolating regions of interest within tissue samples [18, 44, 45]. First, optimisation of the immunostaining of 10 µm skeletal muscle sections was performed using a protocol adapted from Rocha and colleagues [50], to allow visualisation of myonuclei and mitochondria. We then established the working limit of LCM, by determining the smallest possible dissected area that could then be successfully analysed using a qPCR assay targeting mtDNA. We stained human skeletal muscle tissue slices with fluorescently labelled antibodies, targeting mitochondrial encoded cytochrome c oxidase I (MTCOI) and used fluorescent microscopy to confirm the success of the staining (Fig. 2ai and 2bi). We used LCM to dissect regions with a decreasing area and Fig. 2aii shows a BF micrograph after we performed four dissections of 30 µm in diameter. Fluorescence micrographs (Fig. 2aiii) also confirmed the success of the dissections. Similarly, we performed dissections of 5 µm in diameter and imaged the sample before (Fig. 2bi) and after the procedure (Fig. 2bii and 2biii).

A Triplex qPCR protocol from Rygiel and colleagues [49] was adapted for use with subcellular quantities of mtDNA. Using a series of increasingly smaller diameter dissections (*n* = 4 per section) ranging from whole single cells to 5 µm dissections, we were able to show that the reliable working limit of LCM is between 20 and 10 µm (Fig. 2c) and we observed that the mtDNA copy number per microliter (CN/µl is correlated to the dissected area (*r* =  − 0.91, *p* < 0.0001, Fig. 2c). However, beyond 20 µm, mtDNA copy number assessments from repeat sampling showed increased variability and often < 1 copy of mtDNA. Having demonstrated the working limit of LCM, we then investigated if an adaption of the nanobiopsy technology based on a scanning ion conductance microscope (SICM) could be implemented to enable subcellular sampling from human tissue sections with a spatial resolution surpassing the limits of LCM.

**Fig. 3:**
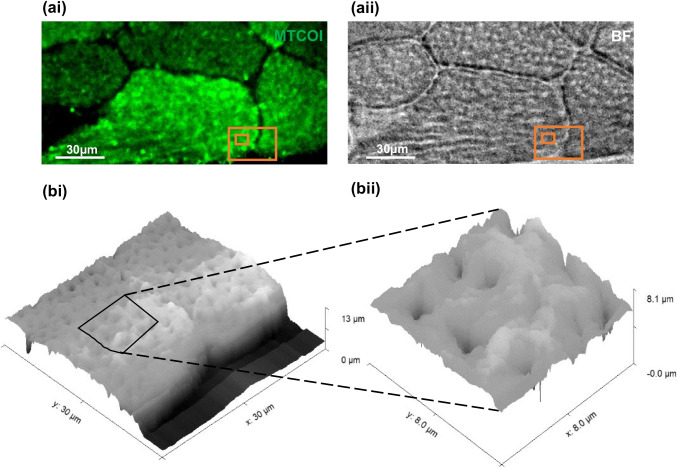
Fluorescent micrograph corresponding to IF labelling of MTCOI (ai) and bright-field micrograph (aii) of a human skeletal tissue slice, with orange boxes delineating the SICM scan areas. SICM topography scans of the region of interest were 30 × 30 μm (bi) and 8 × 8 μm (bii)

SICM enables the topographic mapping of cells and tissues with nanoscale resolution [51]. The SICM setup is mounted on top of an inverted optical microscope to enable simultaneous optical imaging and topographic mapping of the sample of interest. Figure 3 shows the optical imaging of skeletal muscle tissue (panel a) and its topography (panel b) as acquired with the SICM showing myofibrils and sarcoplasm.

### Subcellular mitochondrial biopsy from skeletal muscle fibres

The subcellular biopsy approach relies on a micropipette integrated into an SICM setup. Two twin micropipettes were produced from each capillary tube to specifications adapted from the accompanying manual (Sutter Instrument). An optimised two-line patch pipette programme was used, as follows:

- Line 1—Heat: 700; filament: 4; velocity: 55; delay: 132; pull: 55
- Line 2—Heat: 750; filament: 4; velocity: 50; delay: 127; pull: 70

Scanning electron microscopy (SEM) and electrochemical analyses were used to assess the micropipette geometry and pore size. 

After selecting a region of interest, based on optical imaging, a biopsy can be performed by lowering the micropipette to the selected region by a predetermined depth (2 µm) at a speed of 100 µm/s whilst applying a positive voltage to prevent premature aspiration, which we optimally found to be 200 mV. Our group has previously shown that micro- and nanopipettes can be used to aspirate cytoplasmic content by taking advantage of electrowetting [42, 52] and we investigated if this approach was suitable for the sampling from skeletal muscle fibres.

**Fig. 4:**
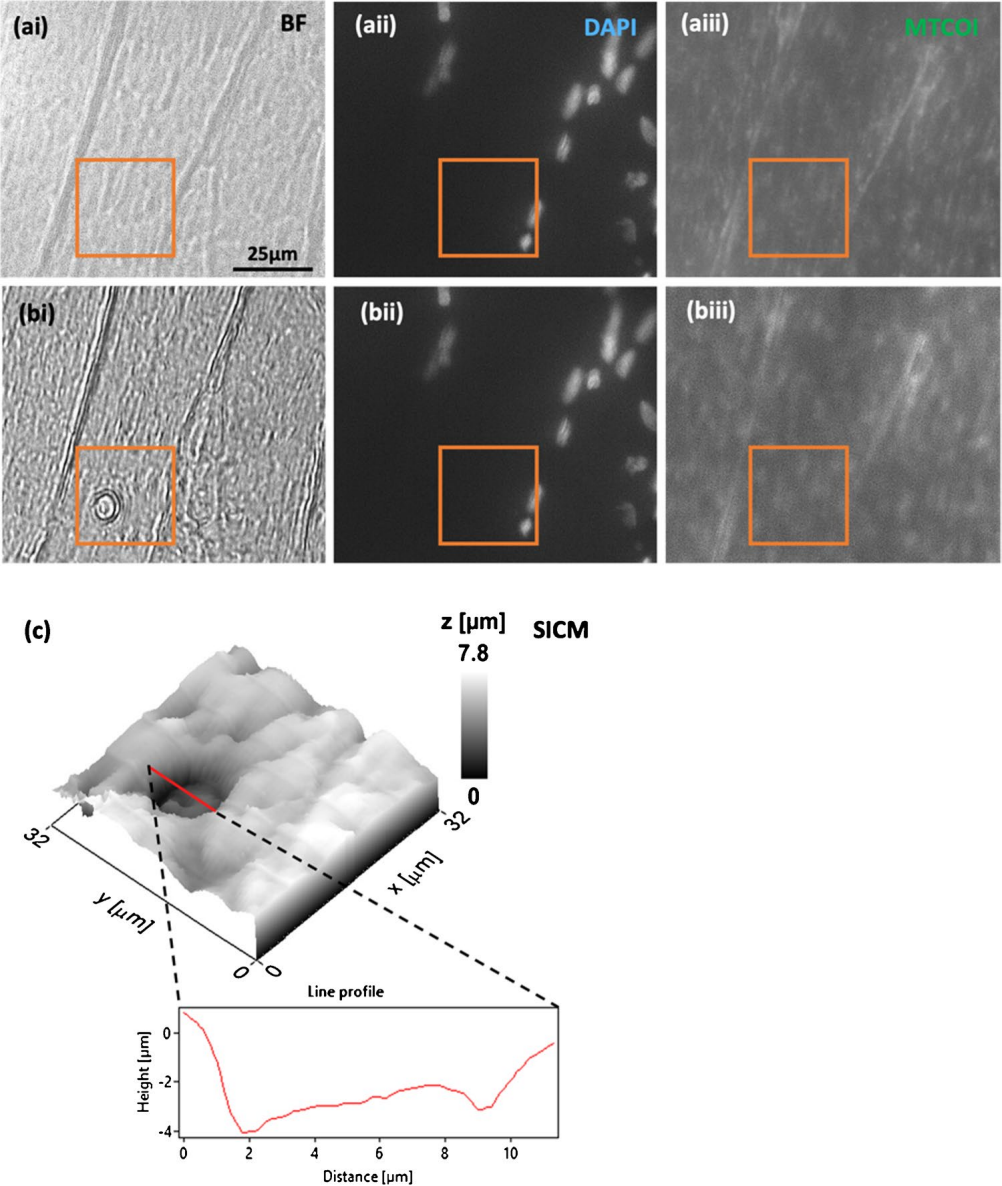
(**ai**–**aiii**) Pre-subcellular biopsy images of skeletal muscle fibres, corresponding to BF (**ai**), myonuclei stained with DAPI (**aii**), and IF staining of MTCOI (**aiii**). (**bi**–**biii**) Post-biopsy images, again BF (**bi**), DAPI (**bii**), and MTCOI IF staining (**biii**). Evidence of successful biopsies is shown in BF (**bi**), but a reduction in MTCOI fluorescence was not shown post-biopsy (**biii**). The relative position of the biopsy to myonuclei is shown relative to DAPI staining, which indicates that biopsies taken are from the intermyofibrillar region and not from the perinuclear region. The biopsy sites in all images (pre- and post-biopsy) were delineated within an orange box. A topographical scan of the area shown within the orange box (32 × 32 µm) further supported the successful biopsy as shown by an indentation in the skeletal muscle fibre, measuring approx. 7 µm in diameter, consistent with the biopsy site observed in BF (**c**)

Figure 4 shows representative optical images (Fig. 4a and b) and a topographical scan (Fig. 4c), before (Fig. 4a) and after (Fig. 4b) the completion of the subcellular biopsy. The BF micrograph post-biopsy (Fig. 4bi) shows what looks like an indentation in the tissue which is confirmed by the post-biopsy SICM scan. The indentation is about 3 µm deep and 7 µm wide as shown in the line profile in Fig. 4c and these dimensions are consistent with the micropipette size employed in this study. Interestingly, we did not observe any noticeable decrease in fluorescence post-biopsy (Fig. 4biii) indicating that a change in fluorescence cannot be used to assess the success of the biopsy as the fluorescent signal could originate from an area underneath the biopsy site.

### Working range of subcellular mitochondrial biopsy

**Fig. 5:**
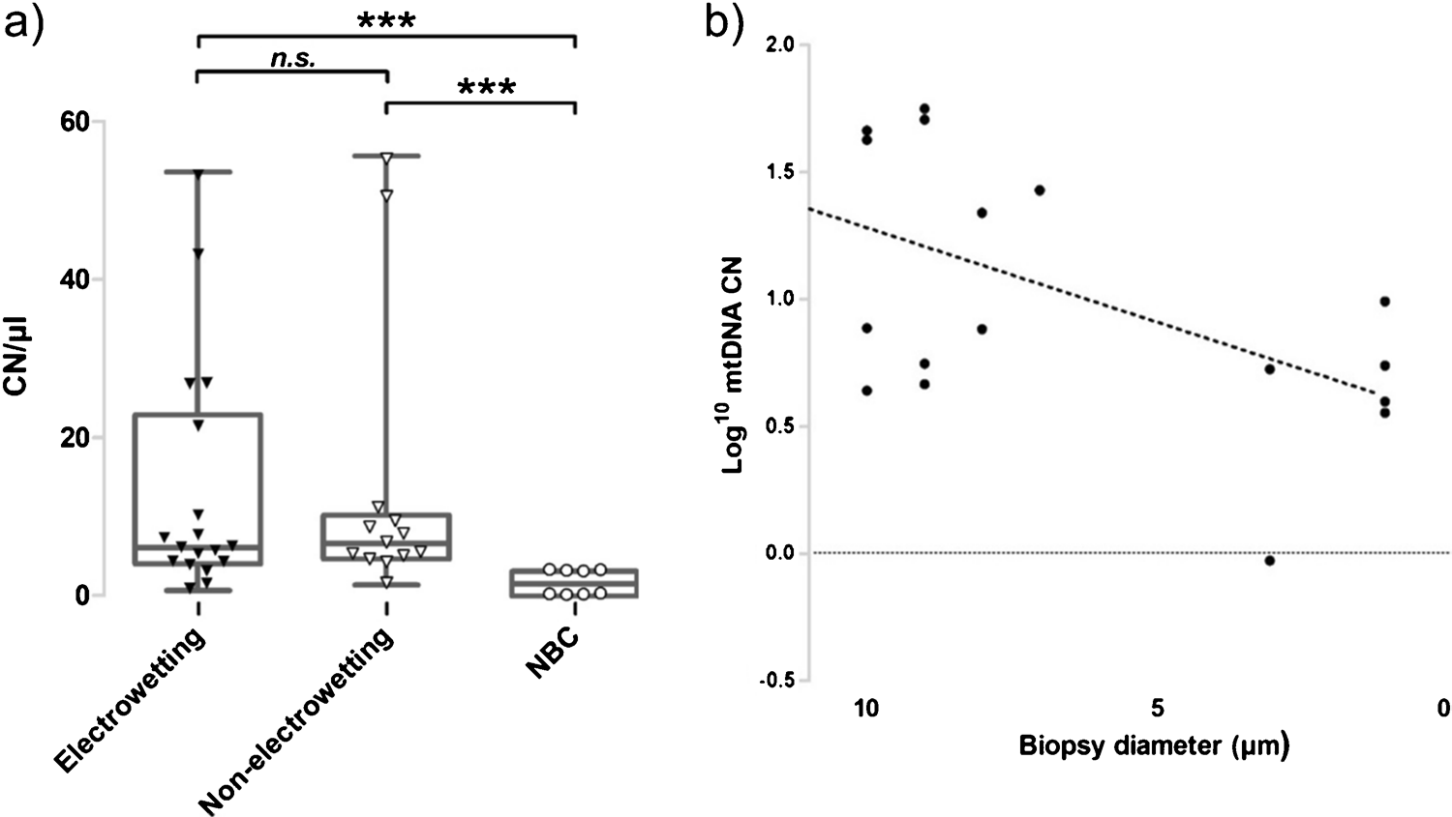
qPCR analysis of the biopsied samples. (**a**) Each data point corresponds a technical triplicate of each biopsy lysate sample. The box plot corresponds to the median and interquartile range of the data points. Data points shown as black triangles correspond to biopsies obtained with electrowetting (*n* = 11; mean = 13.25; median = 6.23) and points depicted as white triangles correspond to non-electrowetting (*n* = 7; mean = 13.55; median = 6.77). Points depicted as white dots correspond to non-biopsy controls (NBC; *n* = 5; mean = 1.71; median = 1.71). A significant difference in CN/µl was observed between biopsies acquired with (electrowetting:* p = *0.0009 < 0.001, Mann–Whitney *U* test) and without electrowetting (non-electrowetting:* p = *0.0006 < 0.001, Mann–Whitney *U* test) compared with NBCs. There was no significant difference in CN/µl observed between biopsies acquired with electrowetting and non-electrowetting (*p* = 0.76, Mann–Whitney *U* test). If potential outliers within the non-electrowetting group are removed (*n* = 6; mean = 6.40; median = 5.53), then a significant difference was still observed between non-electrowetting biopsies and NBCs (*p* < 0.01, Mann–Whitney *U* test). There was also no significant difference between the medians of electrowetting and non-electrowetting biopsy groups (*p* = 0.70, Mann–Whitney *U* test). (**b**) The CN/µl of biopsies (log transformed, *y*-axis) was plotted against biopsy diameter as determined from topographical scans of the biopsy site. The mean micropipette biopsy site diameter was 6.4 µm. Biopsy site diameter and CN/µl value were highly variable and a positive correlation that did not reach significance was observed between biopsy diameter and CN/µl value (*n* = 11, *r* = 0.436, *p* > 0.05)

To compare the effectiveness of subcellular biopsy versus LCM, we performed the same Triplex qPCR mtDNA assay on samples obtained with subcellular biopsy. In addition, we studied if the application of a negative voltage after the micropipette was inserted within the tissue was necessary to control the biopsy (Fig. 5). The application of the negative voltage is necessary to control the electrowetting process but it does not seem to be necessary when a biopsy is performed within a tissue as we did not observe any statistically relevant difference when a biopsy was performed with electrowetting (negative voltage applied in tissue) and non-electrowetting (no voltage applied) (electrowetting mean CN/µl = 13.25; 95% CI = 2.87–24.22; non-electrowetting mean CN/µl = 13.55; 95% CI = 5.76–20.74; Mann–Whitney *U*, *p* > 0.05, Fig. 5). Although electrowetting appears to be critical to the isolation of mitochondria from cultured cells [30, 42], we did not find any experimental evidence that this is necessary to sample from a tissue section. Using subcellular biopsy, we were able to detect ~ 4 mtDNA CN from biopsies with a mean diameter of 6.4 µm, based on SEM and SICM imaging data (*n* = 11; SD = 3.7, Fig. 5). Importantly, in both electrowetting (Mann–Whitney *U*, *p* < 0.001) and non-electrowetting (Mann–Whitney *U*, *p* < 0.001), mtDNA CN values were significantly higher than the non-biopsy controls (mean CN/µl = 1.71; 95% CI = 0.36–3.06).

Similar to LCM (Fig. 2c), mtDNA CN was correlated to the biopsy diameter but a statistically significant correlation was not shown between total biopsy diameter and mtDNA CN (*n* = 11, *r* = 0.436, *p* > 0.05, Fig. 5). A correlation reaching statistical significance was also not observed in either electrowetting (*n* = 6, *r* = 0.571, *p* = 0.06) or non-electrowetting (*n* = 4, *r* = 0.450, *p* = 0.311) biopsy groups.

## Discussion

Subcellular biopsy, as an adaptation of nanobiopsy, is a promising scanning probe technology for use in the isolation of organelles for downstream genomic analysis [27]. Whilst micromanipulation-based techniques and FFM have been previously utilized to acquire mitochondria from cells and tissues [36, 53, 54] and the use of scanning probes have been used to perform topographical scans of human tissue [38, 55, 56], up until now, the utility of scanning probe technologies to aid the successful isolation of organelles from subcellular compartments within human tissue had yet to be demonstrated. We have optimized and utilized subcellular biopsy for the acquisition of mitochondria from skeletal muscle fibres for the first time, with the superior control and sampling resolution offered by scanning probe technologies.

### Working range of laser capture microdissection

A strong correlation was highlighted in the qPCR data between dissection size and mtDNA CN down to dissections of > 20 µm in diameter. For dissection sizes < 20 µm in diameter, a steep drop off in CN was observed. This is likely due to an unsuccessful dissection or due to ablative laser damage to mitochondria, mtDNA, or even the muscle fibres themselves [57, 58]. The latter is supported by observed scorching when areas < 20 µm were circumscribed. This likely shows that the lower operative range of LCM, for isolating organelles and other biomolecules from human tissue, is likely to be in the region of 20 µm. We have demonstrated that the subcellular biopsy has an operative range much lower than this limit. mtDNA CN data obtained from qPCR, coupled with topographical scans of the sampled biopsy sites, demonstrated a linear trend between CN and size, like that observed with LCM. There was no significant correlation between CN and biopsy size; this could be masked by a low *n* number and high variability in biopsy diameter. This variability in biopsy diameter could be attributed to an inconsistent pore size of fabricated pipettes, which serves as a possible limitation of subcellular biopsy. This can be overcome by maintaining consistent environmental conditions, such as temperature, when fabricating pipettes and employing strict exclusion criteria for pipette pull time and pore size, as determined through SEM.

### Optimization of subcellular biopsy in skeletal muscle fibres

The sampling of mitochondria, or other organelles, using scanning probe technologies had previously only been demonstrated in cultured cells [42, 53]. Typically mitochondria from cultured cells tend to be smaller than in tissue [59, 60] and so ensuring the micropipette pore size was sufficiently large to sample mitochondria was the first obstacle. The reported mean maximal diameter of subsarcolemmal (SS) mitochondria in human skeletal muscle tissue is 790 nm, whilst the mean maximal diameter for intermyofibrillar (IMF) mitochondria is 1.2 µm [61]. To avoid introducing sampling bias, micropipettes needed to be large enough to sample mitochondria from the upper range of any mitochondrial subpopulation, whilst still being small enough to sample mitochondrial subpopulations unique to small distinct foci and to minimise the impact to the cellular environment of sampled skeletal muscle fibres [18, 42].

### Subcellular biopsy allows the sampling of mitochondria from skeletal muscle fibres

Successful sampling of mitochondria from human tissues was confirmed by qPCR analysis, where more than two criteria were met. The limit of qPCR assay sensitivity was found to be 4.33 mtDNA CN/µl. The mean CN value of 13.25/µl was obtained with our cookie cutter approach. This is comparable with ~ 6–10 mtDNA copies per mitochondrion [62]. Compared with previous studies reporting isolation of a single mitochondrion [53, 54], it is likely that this corresponds to several mitochondria but crucially our technology enables the sampling from larger or smaller regions of interest by simply adjusting the dimensions of the micropipette and it should theoretically allow the sampling of a single mitochondrion.

qPCR data suggests that electrowetting, whilst not necessarily detrimental, may not be necessary to perform biopsies in skeletal muscle tissue. This observation was exciting because it largely simplifies the operation of the SICM and negates the need for the organic solvent necessary for electrowetting. A key benefit of subcellular biopsy is that the same platform employed for subcellular sampling can be used to acquire topographical scans of the region of interest before and after sampling [56]. Whilst, post-sampling topographical scans were useful in determining the success of sampling, real-time topographical scanning would improve the spatial resolution and speed and allow for automated sampling. Also, double or even multi-barrel micropipettes [52, 63, 64] could be used to allow for improved operations and enable multifunctional measurements. An exciting potential utility of subcellular biopsy, using SICM or other scanning probe systems, could be the acquisition and subsequent transplantation of mitochondria into cultured cells for investigative or therapeutic purposes [36, 65]. The advantage of using an SICM system would be the ability to couple this with the topographical scanning capability of SICM [54, 55].

## Conclusions

In this study, we demonstrated that a micropipette integrated within an SICM can successfully sample mitochondria from human skeletal muscle, or any human tissue, with a spatial resolution higher than the gold standard, LCM. We envision that this technology will enable the isolation and analysis of organelle populations from discrete foci within tissues for downstream molecular and structural analysis.

## Declarations

Control skeletal muscle tissue, from patients undergoing ACL surgery at the Freeman General Hospital (Newcastle, UK), was sourced from the Newcastle Mitochondrial Research Biobank (Newcastle upon Tyne Hospitals NHS Foundation Trust). Ethics approval was granted by The Newcastle and North Tyneside Local Research Ethics Committee (LREC: 12/NE/0395). A pre-print version of this article is available on BioRxiv: 10.1101/2021.11.29.470247.
